# Buparlisib in combination with tamoxifen in pretreated patients with hormone receptor‐positive, HER2‐negative advanced breast cancer molecularly stratified for *PIK3CA* mutations and loss of PTEN expression

**DOI:** 10.1002/cam4.3092

**Published:** 2020-04-30

**Authors:** Anja Welt, Marcel Wiesweg, Sarah Theurer, Wolfgang Abenhardt, Matthias Groschek, Lothar Müller, Jan Schröder, Mitra Tewes, Marco Chiabudini, Karin Potthoff, Agnes Bankfalvi, Norbert Marschner, Martin Schuler, Frank Breitenbücher

**Affiliations:** ^1^ Westdeutsches Tumorzentrum Innere Klinik (Tumorforschung) Universitätsklinikum Essen Universität Duisburg‐Essen Essen Germany; ^2^ Westdeutsches Tumorzentrum Institut für Pathologie Universitätsklinikum Essen Universität Duisburg‐Essen Essen Germany; ^3^ MVZ Onkologie im Elisenhof München Deutschland; ^4^ Hämatologie – Onkologie – Stolberg Stolberg Deutschland; ^5^ Onkologie UnterEms Leer‐Emden‐Papenburg Annenstr. 11 Leer Deutschland; ^6^ Praxis für Hämatologie und Onkologie Mülheim a.d.R Deutschland; ^7^ iOMEDICO AG Freiburg Deutschland; ^8^ Deutsches Konsortium für Translationale Krebsforschung (DKTK) Partnerstandort Universitätsklinikum Essen Essen Deutschland

**Keywords:** breast cancer, buparlisib, clinical trial, phase II, drug therapy, PI3K

## Abstract

The PIKTAM study evaluated the efficacy and safety of the PI3K inhibitor buparlisib in combination with tamoxifen in hormone receptor‐positive (HR^+^), HER2‐negative advanced breast cancer patients after failure of prior endocrine therapy. In this open‐label, single‐arm phase II trial, 25 patients were enrolled in 11 sites in Germany. Patients were stratified according to *PIK3CA* mutation status (tissue and cfDNA from serum samples) and/or loss of PTEN expression. Patients received buparlisib (100 mg) and tamoxifen (20 mg) once daily on a continuous schedule (28‐day cycle) until progression or unacceptable toxicity.

Primary endpoint was overall 6‐month progression‐free survival (PFS) rate. Key secondary endpoints included the 6‐month PFS rate in subpopulations, PFS, overall survival, overall response rate (ORR), disease control rate (DCR), and safety.

Overall, the 6‐month PFS rate was 33.3% (n/N = 7/21, one‐sided 95% CI 16.8‐100) and median PFS was 6.1 (CI 2.6‐10.6) months. The ORR and DCR were 12.5% and 44%. The *PIK3CA*‐mutated subgroup consistently showed the highest 6‐month PFS rate (62.5%, n/N = 5/8), median PFS (8.7 months), ORR (40%), and DCR (80%). No new safety signals emerged. Most common adverse events were gastrointestinal disorders (56%), psychiatric/mood disorders (48%), skin rash/hypersensitivity (44%), cardiovascular (40%), and hepatic (32%) events. The trial was prematurely terminated due to the substantially altered risk‐benefit profile of buparlisib. Nevertheless, *PIK3CA* mutations emerged as a clinically feasible and useful biomarker for combined PI3K inhibition and endocrine therapy in patients with HR^+^ breast cancer. Further biomarker‐stratified studies with isoform‐specific PI3K inhibitors are warranted. *EudraCT No:* 2014‐000599‐24.

## Graphical abstract

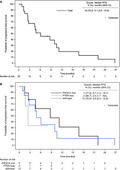

**Novelty and Impact**

This phase II study evaluated the efficacy and safety of buparlisib plus tamoxifen in pretreated patients with advanced HR^+^/HER2^−^ breast cancer, stratified for *PIK3CA* mutations and/or loss of PTEN expression in tumor tissue and circulating blood DNA. A signal for higher buparlisib/tamoxifen activity in patients with *PIK3CA*‐mutated tumors was obtained. Our findings support the value of somatic *PIK3CA* mutations as predictive biomarker for PI3K‐targeting therapies combined with antihormonal treatment in metastatic HR^+^ breast cancer.

## 1 INTRODUCTION

Patients with hormone receptor‐positive (HR^+^) metastatic breast cancer (mBC) receive endocrine therapy (ET) as standard of care.^1^ Although this allows durable disease control, development of resistance is inevitable. Deregulation of the phosphatidylinositol‐4,5‐bisphosphate 3‐kinase/protein kinase b/mechanistic target of rapamycin (PI3K/AKT/mTOR) pathway was identified as important contributor to ET resistance.^2^, ^3^, ^4^, ^5^ Accordingly, strategies targeting mediators of this pathway were developed to prolong ET‐mediated disease control. The mTOR inhibitor everolimus improved PFS and OS when combined with aromatase inhibitors in ET‐pretreated mBC patients (although OS benefit was not statistically significant).^6^, ^7^ Likewise, inhibitors of AKT^8^ and PI3K^9^, ^10^, ^11^, ^12^, ^13^ were clinically explored in this setting. Buparlisib (BKM120), a pan‐class I PI3K inhibitor, was initially studied in several cancer entities including ER^+^ mBC.^9^, ^14^, ^15^, ^16^, ^17^, ^18^ Strong clinical signals were obtained in this entity, in particular when ET was added to buparlisib.^14^ The pivotal studies BELLE‐2^19^ and BELLE‐3^20^ explored the combination of buparlisib with the selective estrogen receptor (ER) degrader fulvestrant^21^, ^22^ in everolimus‐naïve and everolimus‐pretreated patients with ER^+^/HER2^−^ mBC. Several preclinical studies had indicated that PI3K pathway activating mutations could induce sensitivity to PI3K inhibitors^23^, ^24^ and that combining PI3K inhibition and ET in the presence of *PIK3CA* mutations could be a feasible strategy.^25^, ^26^ The BELLE‐2 study was among the first trials to study this concept in the clinical setting. Patients were stratified according to their PI3K pathway activation status determined in tissue samples to test if markers of PI3K/AKT/mTOR pathway deregulation could help to enrich patient populations with a higher chance of deriving clinical benefit from combined ET and PI3K inhibitor therapy.

The study PIKTAM was designed to evaluate the efficacy of buparlisib in combination with the selective ER modulator tamoxifen^27^ in patients with HR^+^/HER2^−^ mBC, patients were stratified for two putative biomarkers of PI3K/AKT/mTOR pathway activation, somatic mutations of *PIK3CA* and loss of phosphatase and tensin homolog (PTEN) expression. While PTEN expression was studied in formalin‐fixed paraffin‐embedded (FFPE) tumor biopsies, somatic *PIK3CA* mutations were assessed in tumor tissues as well as in DNA freely circulating in the bloodstream (cfDNA). Although cfDNA is a mixture of DNA released from normal tissues and tumor DNA, it was shown that somatic hotspot gene mutations can be reliably detected by highly sensitive assay technology.^28^, ^29^, ^30^ We thus planned to compare the individual and combined predictive value of *PIK3CA* mutations for clinical benefit of the study therapy.

The PIKTAM study was originally powered to provide clinically and statistically meaningful signals for the efficacy of buparlisib combined with tamoxifen in the entire study population as well as in the biomarker‐specified strata. While the study was ongoing new safety signals for buparlisib emerged, presented by Dr Baselga at the San Antonio Breast Cancer Symposium in 2015.^31^, ^32^ The steering committee thus decided to terminate the study early based on an altered benefit‐risk assessment. Here, we report the final analysis of safety and efficacy outcomes of PIKTAM. We demonstrate that buparlisib plus tamoxifen is clinically effective in medically pretreated patients with HR^+^/HER2^−^ mBC. We further provide a strong signal that *PIK3CA* mutations may be used as predictive biomarker to enrich the clinically susceptible patient population. These findings add to recently emerging evidence supporting the concept of combining ET with PI3K‐targeting therapy in HR^+^ mBC.^33^

## 2 PATIENTS AND METHODS

### 2.1 Patients

Pre‐ and postmenopausal patients, aged ≥ 18 years, with HR^+^ (ER‐ and/or progesterone receptor (PgR)‐positive), HER2^−^, locally inoperable advanced BC or mBC presenting with measurable or non‐measurable lesions according to Response Evaluation Criteria in Solid Tumors (RECIST) v1.1 and progression following prior ET were eligible if they had a known PI3K pathway biomarker status (activated or nonactivated). Up to 2 prior lines of ET in the metastatic setting (except for tamoxifen) and up to 1 prior palliative chemotherapy were allowed. Prior treatment with tamoxifen was not allowed in the metastatic setting, while this was permitted in the (neo‐)adjuvant setting if treatment was discontinued ≥ 1 year prior to study inclusion. Patients were included if they had an Eastern Cooperative Oncology Group (ECOG) performance status score ≤ 2, adequate bone marrow and organ function, and a fasting plasma glucose (FPG) ≤120 mg/dL or ≤ 6.7 mmol/L and HbA1c ≤ 8.5%. Major exclusion criteria were previous treatment with a PI3K‐, AKT‐, or mTOR‐inhibitor, prior palliative tamoxifen treatment, concurrent treatment with a gonadotropin‐releasing hormone analog, presence of symptomatic CNS metastases, chronic treatment with corticosteroids or other immunosuppressive agents, warfarin or other coumarin‐derived anti‐coagulant, administration of drugs known to be strong inhibitors or inducers of CYP3A, and drugs with a known risk to induce Torsades de Pointes. Patients were excluded if they had a documented history or active morbidity of a major depressive episode, bipolar disorder, other psychiatric disorders, a history of suicidal attempt or ideation, a score ≥ 12 on the Patient Health Questionnaire‐9 (PHQ‐9), selection of option “1”, “2”, or “3” to question 9 (suicide ideation) in the PHQ‐9, a score ≥ 15 on the Generalized Anxiety Disorder 7 (GAD‐7) questionnaire or an anxiety of ≥ grade 3 according to Common Terminology Criteria for Adverse Events (CTCAE), active or previous episodes of pneumonitis, specified cardiac abnormalities, and uncontrolled diabetes mellitus. All relevant administrative bodies and institutional review boards approved the protocol and amendments. The study was conducted in accordance with Good Clinical Practice and the Declaration of Helsinki. All patients provided written informed consent.

### 2.2 Study design and endpoints

The PIKTAM trial (EudraCT No 2014‐000599‐24) was an open‐label, single‐arm, multicenter phase II study with a molecularly stratified parallel cohort design including three molecular strata: “*PIK3CA* mut/ PTEN preserved”, “*PIK3CA* mut or wild‐type (wt)/ PTEN loss”, “*PIK3CA* wt/ PTEN preserved”, in the following denoted as “*PIK3CA*‐mutated subgroup”, “PTEN loss subgroup”, and “wild‐type subgroup”, respectively.

The primary endpoint was the 6‐month PFS rate in the total population. Secondary endpoints in the total population and subgroups (defined by molecular stratification as described above or by PI3K activation status activated vs nonactivated) included the 6‐month PFS rate, PFS, overall survival (OS), overall response rate (ORR), disease control rate (DCR), as well as safety.

### 2.3 Biomarker analysis

Biomarker analysis was performed on archival formalin‐fixed tumor biopsy of primary tumor or metastasis in which PI3K activation was defined as at least one mutation in *PIK3CA* coding exon 9 or 20 as defined by Sanger sequencing and/or loss of PTEN expression, analyzed by immunohistochemistry as described previously.^34^

Additionally, *PIK3CA* activating mutations of coding exon 20 (leading to amino acid change H1047R) were also assessed in cfDNA from serum samples by a modified real‐time polymerase chain reaction (RT‐PCR) as described elsewhere ^35^ using gene‐specific primers (PI3K A: ATCCAgAgTgAgCTTTCATTTTCTC, PI3K F: TCgAAAgACCCTAgCCTTAgATAAA), mutation‐specific fluorescent probes (Anchor PI3K: gTggAAgATCCATCCATTTTTgTTgTCC—FL, Sensor MutLC640‐gCCACCATgACgTgCATC—PH), and wild‐type–specific locked nucleic acids (PIK3 LNA 5′+C + A+C + C+A + T+g + A+T + g+T + g+C + A+T‐‐NH 2).

Serum samples were collected at screening, at Day 1 of cycle 4, and at end of treatment. Patients for whom a mutation in coding exon 20 was detected in at least one serum sample were considered as “*PIK3CA* cfDNA mutation” in contrast to the remaining patients classified as “*PIK3CA* cfDNA wild‐type”. Mutations in coding exon 9 were not analyzed in cfDNA and therefore patients with mutations of coding exon 9 detected in tumor samples were excluded from analysis.

### 2.4 Study medication

Patients were treated with buparlisib (100 mg) and tamoxifen (20 mg) orally once daily on a continuous schedule (28‐day cycle) until progression, intolerable toxicity, or withdrawal of consent. Buparlisib hard gelatin capsules (10 and 50 mg) were provided by the funder of the study. Tamoxifen was prescribed by investigators.

Dose adjustments were permitted to facilitate continuation of study treatment for patients who did not tolerate the protocol‐specified dosing schedule. Two dose reduction levels for buparlisib, 80 and 60 mg/d, were allowed, as well as interruption up to 28 days.

### 2.5 Tumor assessment

The overall response was to be evaluated by the investigator in accordance with RECIST v1.1^36^ every 12 weeks (±7 days) until progressive disease (PD) or start of new antineoplastic therapy. Patients with nonmeasurable lesions only were assessed for complete remission (CR), PD, and non‐CR/non‐PD, while patients with at least one measurable lesion at enrolment were assessed for CR, partial remission (PR), stable disease (SD), and PD and included in the analysis of ORR and DCR. SD as best response was only included in the DCR if the respective tumor assessment was performed at least 12 weeks (minus 7 days) after the treatment starts.

### 2.6 Safety

The safety evaluation included adverse event (AE) assessment, laboratory parameters, ECG, and cardiac imaging, as well as incidence and severity of mood alterations and depressive episodes, assessed by the PHQ‐9 and GAD‐7 questionnaires.

#### 2.6.1 Treatment‐emergent adverse events

Any treatment‐emergent adverse event (TEAE) was to be recorded by the investigator from day of first administration of study treatment until 30 days after end of treatment (EOT; treatment phase). AEs were coded by using the Medical Dictionary for Regulatory Activities (MedDRA) v20.0 and graded by the investigator according to NCI‐CTCAE v4.03.^37^ TEAEs were classified as related if a relationship to buparlisib was documented by the investigator.

#### 2.6.2 Questionnaires

The PHQ‐9 and GAD‐7 are self‐reporting tools designed to detect the presence and severity of depression, comprising 9 depressive symptom criteria including suicide ideation,^38^, ^39^ or anxiety, comprising 7 questions covering mood conditions,^40^, ^41^, ^42^ respectively. The severity of the mood disorder is rated by a questionnaire‐specific scale, in which higher values in the scoring systems of PHQ‐9 and GAD‐7 are an indicator of a more pronounced depression or anxiety, respectively.^38^, ^40^

The PHQ‐9 and GAD‐7 questionnaires were analyzed for the safety set (SAF) at baseline (≤8 days prior to treatment start or on cycle 1 day 1) and throughout the course of the trial at specific time points, ie, in cycle 1 (day 15), cycle 2 (day 1 and day 15), subsequent cycles (each on day 1), and at EOT. The scoring system for each questionnaire was categorized into 4 severity grades as follows: “none” (PHQ‐9 and GAD‐7: total score 0‐4), “mild” (PHQ‐9 and GAD‐7: total score 5‐9), “moderate” (PHQ‐9: total score 10‐19; GAD‐7 total score 10‐14), and “severe” (PHQ‐9: total score 20‐27; GAD‐7 total score: 15‐21). A patient presenting with suicide ideation had to interrupt study treatment and be referred for psychiatric consultation. If a patient did not respond to question 9 dealing with suicide ideation (PHQ‐9), assessment of suicidal ideation was required.

### 2.7 Statistical analysis

The null hypothesis for the 6‐month PFS rate stated that *p*_0_ ≤ 0.400, the alternative hypothesis stated that *p*_1_ ≥ 0.54. Employing a one‐sided exact binomial test, a sample size of 84 patients ensured a power of 80% at a significance level of 5%. A total sample size of 99 patients (33 per stratum) was required including dropouts (15 patients).

The mITT was used to assess the primary endpoint and response rates and was defined as all patients who had received at least one dose of buparlisib and who had a 6‐month tumor assessment (defined as tumor assessment after 24 weeks ± 7 days) unless patients had progressed or died before month 6. Patients without 6 months tumor assessment but any later assessment with a response of CR, PR, SD, or non‐CR/non‐PD were included in the analysis of the primary endpoint. Patients discontinuing the study prior to the 6 months tumor assessment for reasons other than progression or death were not included in the analysis of the primary endpoint. Best response, ORR, and DCR were analyzed for patients with measurable disease within the mITT population. Secondary endpoint analysis, initially planned for the mITT, was performed using the SAF for PFS and OS due to low patient numbers. The SAF comprised all patients who had received at least one dose of study medication and who had at least one postbaseline safety assessment (AE documented or “no AE occurred” documented at EOT). The SAF was used in all safety analyses (including treatment duration and EOT reasons) and served as the analytical population for assessment of PFS and OS including 1‐ and 2‐year overall survival rates (OSRs) as well as baseline demographics and clinical characteristics of patients.

Secondary endpoints were analyzed in the total population (SAF or mITT as described before) and the three molecular stratification subgroups. An additional analysis was performed, in which the two patient subgroups “*PIK3CA* mut/ PTEN preserved” and “*PIK3CA* mut or wt/ PTEN loss” were combined into one subgroup regarded as “PI3K‐activated” subgroup. PFS and OS were estimated by using the Kaplan‐Meier method.^43^

Based on former AE reporting in context of buparlisib treatment, the reported AE terms were additionally clustered into prespecified specific safety event categories (SECs). These included AEs concerning hyperglycemia, mood disorders, liver toxicity, skin rash and hypersensitivity, posterior reversible encephalopathy syndrome, gastrointestinal events, lung toxicity/pneumonitis, and cardiovascular events. The assignment of a TEAE to a SEC was based on the reported term for respective AE. For SEC analysis, only the first occurrence of a SEC per patient was included in the analysis.

Dose intensity, number of dose modifications, and reasons were summarized for the SAF. The relative dose intensity equaled the cumulative dose per cycle divided by the planned dose per cycle. In case a patient did receive neither of the study treatments in the last documented cycle, the cycle was excluded from the analysis of the respective patient. Dose modifications were counted only once per patient and type of modification.

Exploratory analyses included evaluation of PFS and OS by *PIK3CA* mutation status as determined by cfDNA analyses as described above to assess their predictive value of clinical benefit in advanced HR^+^/HER2^−^ BC. Analyses by cfDNA status were conducted based on the SAF for patients with determinable cfDNA status. If not otherwise stated, confidence intervals stated refer to 95% CI.

## 3 RESULTS

### 3.1 Disposition of patients and patient characteristics at baseline

**FIGURE 1:**
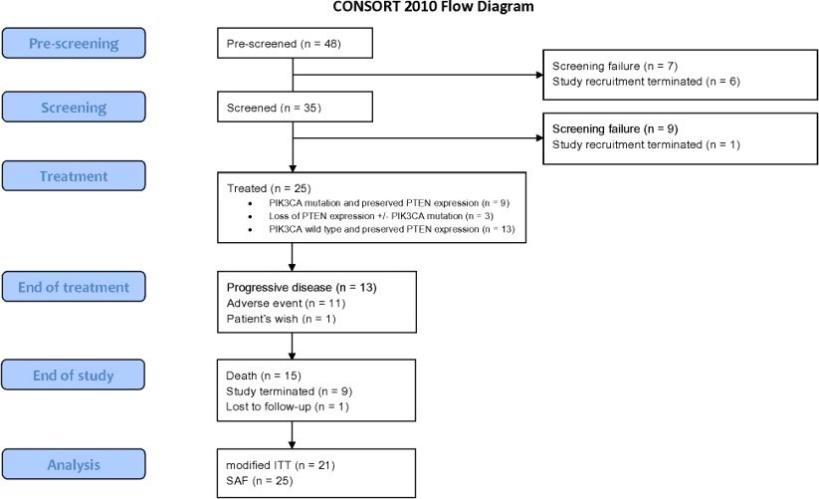
CONSORT flow diagram. The reason “adverse event” includes both inacceptable toxicity and (serious) adverse events. After prescreening for identification of biomarker status, patients were screened for eligibility and assigned to the respective subgroup by molecular stratification. Study recruitment was terminated by sponsor due to reevaluation of the risk‐benefit profile of buparlisib. Abbreviations: n: number (of events); *PIK3CA*: Phosphatidylinositol 4,5‐bisphosphate 3‐kinase catalytic subunit alpha isoform; PTEN, Phosphatase and tensin homolog; ITT, intention‐to‐treat; SAF, safety set

The PIKTAM study started in December 2014 (first patient in) and ended in October 2017 (last patient out). This study was initially planned to enroll 99 patients. Due to reevaluation of the risk‐benefit profile of buparlisib, based on the outcome of the BELLE‐2 study, patient recruitment in the PIKTAM trial was prematurely terminated by the sponsor in January 2016. Of 48 screened patients, 25 patients with known *PIK3CA*/ PTEN biomarker status were enrolled in 11 centers in Germany (Figure 1). The mITT and SAF population comprised 21 and 25 patients, respectively.

All enrolled patients received study treatment and were stratified into three biomarker‐defined subgroups based on analysis of archival tumor tissue: *PIK3CA*‐mutated subgroup (mITT N = 8; SAF N = 9), PTEN loss subgroup (mITT N = 2; SAF N = 3,) and wild‐type subgroup (mITT N = 11; SAF N = 13) (please refer to patients and methods section for detail on the molecular strata). In a subgroup of patients, *PIK3CA* mutations were studied in cfDNA. Since the assay only covered *PIK3CA* coding exon 20, patients with known mutations in *PIK3CA* coding exon 9 (n = 4) were excluded from this analysis. Another three patients were excluded due to missing cfDNA status. Hence, the cfDNA analysis population (mITT N = 15; SAF N = 18) was subgrouped in “*PIK3CA* cfDNA mutation” (mITT N = 4; SAF N = 4) and “*PIK3CA* cfDNA wild‐type” (mITT N = 11; SAF N = 14) patients. Details on mutation status analysis are provided in Supplements 1 and 2.

Baseline demographics, main tumor characteristics, and treatment history of the patients are detailed in Table 1 for the total population and per biomarker subgroup (SAF). In the total population, median age was 62.9 years, 72% had ECOG 0, and 84% presented with visceral disease. Overall, 80% of all patients had received at least one prior line of ET, of whom, 72% had received at least one prior line of therapy with aromatase inhibitors and 24% at least one previous line of therapy with fulvestrant in the metastatic setting. In total, 24% of all patients had received 2 prior lines of palliative ET, while 32% of patients had been subjected to palliative chemotherapy.

**TABLE 1:** Demographics and clinical characteristics of patients at baseline in total population and by biomarker stratification group (SAF)

		*PIK3CA* mut (N = 9)	PTEN loss^a^(N = 3)	Wild‐type (N = 13)	Total population (N = 25)
Median age^b^, ^a^, years (min‐max)		63.6 (57.8‐80.7)	69.0 (62.5‐71.7)	61.8 (49.0‐80.6)	62.9 (49.0‐80.7)
BMI [kg/m^2^], median (min‐max)		27.9 (17.2‐34.8)	21.8 (21.2‐27.7)	24.0 (19.7‐32.8)	24.2 (17.2‐34.8)
ECOG performance status, n (%)	0	7 (77.8)	1 (33.3)	10 (76.9)	18 (72.0)
	1	2 (22.2)	2 (66.7)	3 (23.1)	7 (28.0)
	2	0 (0.0)	0 (0.0)	0 (0.0)	0 (0.0)
Hormone receptor status, n (%)	ER positive	9 (100.0)	3 (100.0)	13 (100.0)	25 (100.0)
	PgR positive	9 (100.0)	3 (100.0)	6 (46.2)	18 (72.0)
*PIK3CA* mutation status, by archival tumor tissue, n (%)^a^	Mutated coding exon 9	4 (44.4)	0 (0)	0 (0)	4 (16.0)
	Mutated coding exon 20	5 (55.6)	0 (0)	0 (0)	5 (20.0)
PTEN mutation status, n (%)	PTEN preserved	9 (100.0)	0 (0)	13 (100.0)	22 (88.0)
	PTEN loss	0 (0)	3 (100.0)	0 (0)	3 (12.0)
Tumor status at primary diagnosis—M0/M1	M0	5 (55.6)	2 (66.7)	10 (76.9)	17 (68.0)
	M1	4 (44.4)	1 (33.3)	3 (23.1)	8 (32.0)
DFI, years [median (min‐max)]^e^		13.7 (2.4‐15.1)	14.4 (14.4‐14.4)	8.2 (2.1‐16.1)	10.6 (2.1‐16.1)
Median time since primary diagnosis to date of first study treatment, years (min‐max)		6.1 (1.6‐26.9)	4.9 (0.9‐17.9)	8.8 (0.9‐21.4)	7.8 (0.9‐26.9)
Visceral disease present, n (%)		8 (88.9)	3 (100.0)	10 (76.9)	21 (84.0)
Metastatic sites^c^, ^d^	Bone	9 (100.0)	2 (66.7)	8 (61.5)	19 (76.0)
	Liver	5 (55.6)	1 (33.3)	8 (61.5)	14 (56.0)
	Lung	3 (33.3)	2 (66.7)	3 (23.1)	8 (32.0)
Prior (neo‐)adjuvant therapy^c^, n (%)	ET	4 (44.4)	2 (66.7)	9 (69.2)	15 (60.0)
	CHT	4 (44.4)	2 (66.7)	6 (46.2)	12 (48.0)
Patients with prior palliative treatment, n (%)	Palliative	9 (100.0)	2 (66.7)	11 (84.6)	22 (88.0)
Prior lines of ET in metastatic setting, n (%)	0	0 (0.0)	1 (33.3)	4 (30.8)	5 (20.0)
	1	7 (77.8)	2 (66.7)	5 (38.5)	14 (56.0)
	2	2 (22.2)	0 (0.0)	4 (30.8)	6 (24.0)
Prior ET in metastatic setting^c^, n (%)	Fulvestrant	3 (33.3)	0 (0.0)	3 (23.1)	6 (24.0)
	AI	8 (88.9)	1 (33.3)	9 (69.2)	18 (72.0)
Prior CHT in metastatic setting, n (%)		2 (22.2)	1 (33.3)	5 (38.5)	8 (32.0)

Note

Demographics and clinical characteristics at baseline in the total population and in the prespecified subgroups: *PIK3CA* mut = “*PIK3CA* mut/ PTEN preserved”; PTEN loss =  “*PIK3CA* mut or wt/ PTEN loss”; wild‐type =  “*PIK3CA* wt/ PTEN preserved”.

Abbreviations: AI, aromatase inhibitors; BMI, body mass index; CHT, chemotherapy ECOG, Eastern Cooperative Oncology Group; ER, estrogen receptor; ET, endocrine therapy DFI, disease‐free interval; mut, mutant; PI3K, Phosphatidylinositol‐4,5‐bisphosphate 3‐kinase; PgR, Progesterone receptor; *PIK3CA*, Phosphatidylinositol‐4,5‐bisphosphate 3‐kinase, catalytic subunit alpha; PTEN, Phosphatase and tensin homolog; SAF, safety set; wt, wild‐type.

^a^ *PIK3CA* mutations in coding exons 9 and 20 were mutually exclusive. No patients with mutations in *PIK3CA* presented additionally PTEN loss, ie, all patients with *PIK3CA* mutations fell into the ‘*PIK3CA* mut/ PTEN preserved’ stratification group.

^b^ On date of informed consent.

^c^ More than one entry per patient possible.

^d^ Presented here are metastatic sites at enrolment that showed in ≥30% of total SAF population.

^e^ Median disease‐free interval was defined as the time from the last R0 resection of the primary tumor to the date of first local relapse or occurrence of distant metastases. DFI was only calculated for patients with M0 status at primary diagnosis and R0 resection (total population N = 13; *PIK3CA* mut/ PTEN preserved N = 4; *PIK3CA* mut or wt/ PTEN loss N = 1; *PIK3CA* wt/ PTEN preserved N = 8).

## 4 EFFICACY

### 4.1 Primary endpoint: 6‐month PFS

In the total mITT population (N = 21), 7 patients (33.33%, one‐sided CI 16.82‐100) were progression free at 6 months (Table 2; one‐sided exact binomial test *P* = .800).

**TABLE 2:** PFS rate in total population by biomarker stratification group (tumor tissue) and by *PIK3CA* cfDNA mutation status (mITT population)

Biomarker stratification group	*PIK3CA* mut (N = 8)	PTEN loss (N = 2)	Wild‐type (N = 11)	Total population (N = 21)
6‐mo PFS rate, n (%)	5 (62.5)	0 (0.0)	2 (18.2)	7 (33.3)
One‐sided 95% CI	28.9‐100	0.00‐100	3.3‐100	16.8‐100
One‐sided *P*‐value	0.174	1.000	0.970	0.800

*PIK3CA* cfDNA mutation status	*PIK3CA* cfDNA mutation (N = 4)	*PIK3CA* cfDNA wild‐type (N = 11)	Total population (N = 15)
6‐mo PFS rate, n (%)	2 (50.0)	4 (36.4)	6 (40.0)
One‐sided 95% CI	9.8‐100	13.5‐100	19.1‐100

Note

PFS rate in the total population, in the prespecified subgroups: *PIK3CA* mut = “*PIK3CA* mut/ PTEN preserved”; PTEN loss = “*PIK3CA* mut or wt/ PTEN loss”; wild‐type = “*PIK3CA* wt/ PTEN preserved” and by *PIK3CA* cfDNA mutation status. *P*‐values refer to a one‐sided exact binomial test testing the null hypothesis 6‐mo PFS rate ≤ 0.4.

Abbreviations: CI, confidence interval; mITT, modified intention‐to‐treat; mut, mutant; PFS, progression‐free survival; PI3K, Phosphatidylinositol‐4,5‐bisphosphate 3‐kinase; *PIK3CA*, Phosphatidylinositol‐4,5‐bisphosphate 3‐kinase, catalytic subunit alpha; PTEN, Phosphatase and tensin homolog; wt, wild‐type

### 4.2 Secondary endpoints

#### 4.2.1 Six‐month PFS rate in subgroups

Regarding tumor tissue‐based subgroups, the 6‐month PFS rate was highest (62.5%; one‐sided CI 28.92‐100) in the *PIK3CA*‐mutated subgroup (N = 8) and lowest (0%; one‐sided CI 0.0‐100) in the PTEN loss subgroup (N = 2). In the wild‐type subgroup (N = 11), it was 18.18% (one‐sided CI 3.33‐100) (Supplement 3).

Stratification by “PI3K activation status” (“activated” denotes *PIK3CA* mutation, or PTEN loss, or both) in tumor biopsies yielded a 6‐month PFS rate of 50.00% (one‐sided CI 22.24‐100) in the “PI3K activated” subgroup (N = 10) and 18.18% (one‐sided CI 3.33‐100) in the “PI3K non‐activated” subgroup (*PIK3CA* wt and PTEN preserved) (N = 11).

The cohort with available *PIK3CA* cfDNA mutation status (N = 15) had an overall 6‐month PFS rate of 40% (one‐sided CI 19.09‐100) with 50% (one‐sided CI 9.76‐100) in the “*PIK3CA* cfDNA mutation” (N = 4) and 36.36% (one‐sided CI 13.51‐100) in the “*PIK3CA* cfDNA wild‐type” (N = 11) subgroup.

#### 4.2.2 Progression‐free survival

**FIGURE 2:**
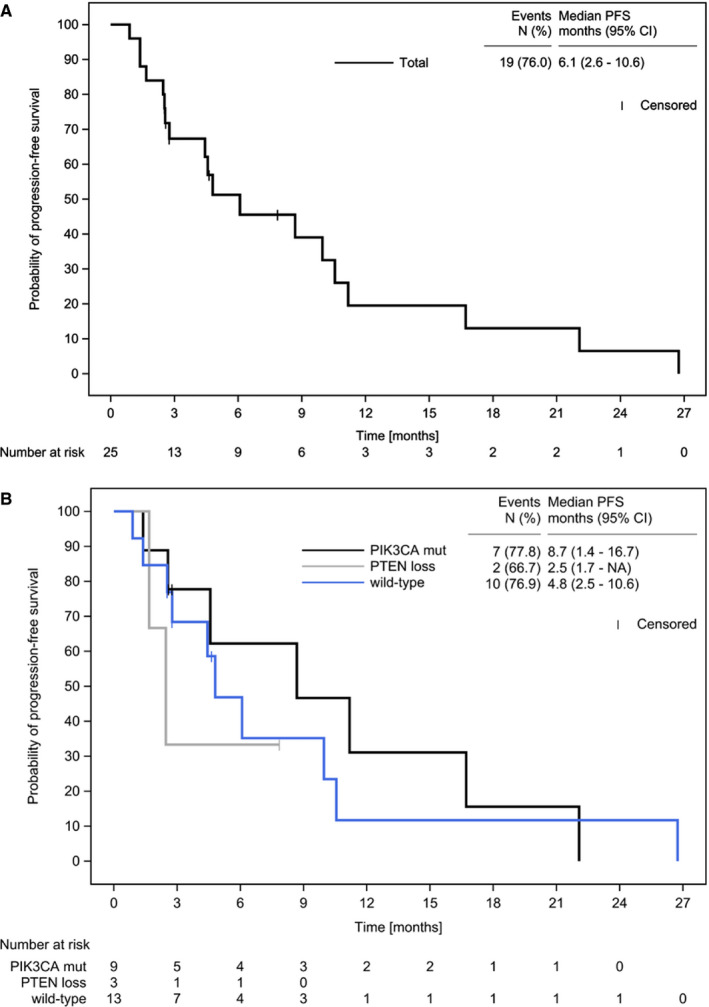
Kaplan‐Meier Estimate of Progression‐Free Survival. Displayed are progression‐free survival in the total patient population (A) and in the prespecified subgroups: *PIK3CA* mut = “*PIK3CA* mut/ PTEN preserved”; PTEN loss = “*PIK3CA* mut or wt/ PTEN loss”; wild‐type = “*PIK3CA* wt/ PTEN preserved” (B) in the SAF population. Abbreviations: CI, confidence interval; n, number (of events); NA, not reached; PFS, progression‐free survival

Median PFS was 6.1 months (CI 2.6‐10.6) in the total population (N = 25) (Figure 2A, Table 3), 8.7 months (CI 1.4‐16.7) in the *PIK3CA* mutated subgroup (N = 9), 2.5 months (CI 1.7‐NA) in the PTEN loss subgroup (N = 3), and 4.8 months (CI 2.5‐10.6) in the wild‐type subgroup (N = 13) (Figure 2B).

**TABLE 3:** Efficacy analysis in the total population and in prespecified, biomarker‐stratified subgroups. (A) PFS (SAF), (B) OS (SAF), and (C) Response rates (mITT population)

	*PIK3CA* mut (N = 9)	PTEN loss (N = 3)	Wild‐type (N = 13)	Total population (N = 25)
(A) Progression‐free survival				
Events, n (%)	7 (77.8)	2 (66.7)	10 (76.9)	19 (76.0)
Censored, n (%)	2 (22.2)	1 (33.3)	3 (23.1)	6 (24.0)
25%‐Quantile, months (95% CI)	4.6 (1.4‐11.2)	1.7 (1.7‐NA)	2.8 (0.9‐4.8)	2.6 (0.9‐4.6)
50%‐Quantile (Median), months (95% CI)	8.7 (1.4‐16.7)	2.5 (1.7‐NA)	4.8 (2.5‐10.6)	6.1 (2.6‐10.6)
75%‐Quantile, months (95% CI)	16.7 (4.6‐22.1)	NA (1.7‐NA)	10.0 (4.8‐26.7)	11.2 (6.1‐26.7)
(B) Overall survival				
Events, n (%)	6 (66.7)	2 (66.7)	7 (53.8)	15 (60.0)
Censored, n (%)	3 (33.3)	1 (33.3)	6 (46.2)	10 (40.0)
25%‐Quartile, months (95% CI)	13.9 (5.7‐25.1)	1.7 (1.7‐NA)	12.3 (4.7‐23.8)	12.6 (1.7‐22.3)
50%‐Quartile (Median), months (95% CI)	25.1 (5.7‐NA)	24.0 (1.7‐NA)	23.8 (10.6‐NA)	24.0 (13.9‐NA)
75%‐Quartile, months (95% CI)	25.5 (15.0‐NA)	NA (1.7‐NA)	NA (22.3‐NA)	NA (24.0‐NA)

	*PIK3CA* mut (N = 5)	PTEN loss (N = 2)	Wild‐type (N = 9)	Total population (N = 16)
(C) Response rates (RECIST v1.1)				
Best response				
CR, n (%), 95% CI	0 (0.0), 0.0‐52.2	0 (0.0), 0.0‐84.2	0 (0.0), 0.0‐33.6	0 (0.0), 0.0‐20.6
PR, n (%), 95% CI	2 (40.0), 5.3‐85.3	0 (0.0), 0.0‐84.2	0 (0.0), 0.0‐33.6	2 (12.5), 1.6‐38.3
SD, n (%), 95% CI	3 (60.0), 14.7‐94.7	0 (0.0), 0.0‐84.2	5 (55.6), 21.2‐86.3	8 (50.0), 24.7‐75.3
PD, n (%), 95% CI	0 (0.0), 0.0‐52.2	1 (50.0), 1.3‐98.7	4 (44.4), 13.7‐78.8	5 (31.3), 11.0‐58.7
Not done^b^, ^a^, n (%), 95% CI	0 (0.0), 0.0‐52.2	1 (50.0), 1.3‐98.7	0 (0.0), 0.0‐33.6	1 (6.3), 0.2‐30.2
ORR, n (%), 95% CI	2 (40.0), 5.3‐85.3	0 (0.0), 0.0‐84.2	0 (0.0), 0.0‐33.6	2 (12.5), 1.6‐38.3
DCR, n (%), 95% CI	4 (80.0), 28.4‐99.5	0 (0.0), 0.0‐84.2	3 (33.3), 7.5‐70.1	7 (43.8), 19.8‐70.1

Note

Progression‐free survival, overall survival, and response rates (RECIST v1.1) in the total population and in the prespecified subgroups: *PIK3CA* mut = “*PIK3CA* mut/ PTEN preserved”; PTEN loss = “*PIK3CA* mut or wt/ PTEN loss”; wild‐type = “*PIK3CA* wt/ PTEN preserved”. (A) For PFS, patients without progression or death were censored at the date of last tumor assessment or at the start date of a subsequent antineoplastic therapy. (B) For OS, patients without documented date of death at the end of study were right censored at the last date known to be alive. (C) Response evaluation included all tumor evaluations from date of first study drug administration until the 6‐mo tumor assessment. Only patients with measurable lesions at baseline were included in the analysis. A best response “stable disease” (SD) was included in the DCR only if the respective tumor assessment was performed at least 12 wks (minus 7 d) after treatment start.

Abbreviations: CI, confidence interval; CR, complete response; DCR, disease control rate; mITT, modified intention‐to‐treat; mut, mutant; ORR, overall response rate; PD, progressive disease; PI3K, Phosphatidylinositol‐4,5‐bisphosphate 3‐kinase; *PIK3CA*, Phosphatidylinositol‐4,5‐bisphosphate 3‐kinase, catalytic subunit alpha; PR, partial response; PTEN, Phosphatase and tensin homolog; SAF, safety set; SD, stable disease; wt, wild‐type.

^a^ There was no further assessment after baseline assessment.

Stratification by “PI3K activation status” yielded a median PFS of 8.7 months (CI 1.7‐16.7) in the “PI3K activated” subgroup (N = 12) and 4.8 months (CI 2.5‐10.6) in the “PI3K non‐activated” subgroup (N = 13).

*PIK3CA* cfDNA mutation status analysis revealed a median PFS of 10 months (CI 2.8‐16.7), in the total population (N = 18), 9.7 months (CI 2.6‐22.1) in the “*PIK3CA* cfDNA mutation” subgroup (N = 4) and 10 months (CI 2.5‐11.2) in the “*PIK3CA* cfDNA wild‐type” subgroup (N = 14). There was good concordance between the mutation status derived from the tumor sample and the mutation status of the cfDNA (Cohens kappa 0.56 [95% CI: 0.12‐1.00]).

#### 4.2.3 Overall survival

Median OS in the total population (N = 25) was 24.0 months (CI 13.9‐NA) (Table 3, Supplement 4). The 1 and 2‐year OSRs were 80% (CI 58.4‐91.1) and 50.6% (CI 29.5‐68.5), respectively. Median OS was 25.1 months (CI 5.7‐NA), 24.0 months (CI 1.7‐NA), and 23.8 months (CI 10.6‐NA), 1‐year OSRs were 88.9% (CI 43.3‐98.4), 66.7% (CI 5.4‐94.5), and 76.9% (CI 44.2‐91.9), 2‐year OSRs were 55.6% (CI 20.4‐80.5), 66.7% (CI 5.4‐94.5), and 44.9% (CI 17.7‐69.0) in the *PIK3CA*‐mutated subgroup (N = 9), PTEN loss subgroup (N = 3), and the wild‐type subgroup (N = 13), respectively. Similar results were observed for “PI3K activated” vs “non‐activated” subgroups as well as for subgroups according to cfDNA mutation status (for details please refer to Supplement 4).

### 4.3 Response rates

None of the patients of the mITT with measurable disease (N = 16) was reported with a CR (0%, n = 0, CI 0.0‐20.6) as best response. PR was reported in 12.5% (n = 2, CI 1.6‐38.3) of patients and 50% (n = 8) were documented with SD (CI 24.7‐75.3), whereas 31.3% (n = 5) had PD (CI 11.0‐58.7) as best response. ORR was 12.5% (n = 2, CI 1.6‐38.3), 40% (n = 2, CI 5.3‐85.3), 0% (n = 0, CI 0.0‐84.2), and 0% (n = 0, CI 0.0‐33.6) and DCR was 43.8% (n = 7, CI 19.8‐70.1), 80% (n = 4, CI 28.4‐99.5), 0% (n = 0, CI 0.0‐84.2), and 33.3% (n = 3, CI 7.5‐70.1) for total population, the *PIK3CA*‐mutated subgroup (N = 5), the PTEN loss subgroup (N = 2), and the wild‐type subgroup (N = 9) (please refer to Supplement 5 for other subgroup analyses).

### 4.4 Safety

#### 4.4.1 Dose intensity, dose and treatment modifications, treatment duration, and discontinuation

In the total population, the median relative dose intensity of buparlisib was 76.4% (min‐max 30.4%‐100%) and 96.4% (30.4%‐110.7%) for tamoxifen. Regarding buparlisib treatment, median treatment duration was 2.6 months (min‐max 0.3‐26.7 months). Seven patients (28%) were subjected to dose modifications, 14 patients (56%) were reported with a treatment interruption and 4 patients (16%) missed an entire treatment cycle. The main reason for buparlisib treatment modification was toxicity (n = 14, 56%). Referring to tamoxifen treatment, median treatment duration was 4.1 months (min‐max 0.4‐26.7 months), 1 patient (4%) was subjected to a dose modification, and 9 patients (36%) were reported with a treatment interruption. The main reason for tamoxifen treatment modification also was toxicity (n = 7, 28%).

Main reasons for permanent treatment discontinuation were disease progression (n = 13, 52%) and toxicity (n = 11, 44.0%). Adverse events (any grade) leading to treatment discontinuation mostly concerned psychiatric disorders (20%), gastrointestinal disorders (12%), nervous system disorders (12%), as well as skin and subcutaneous tissue disorders (12%).

#### 4.4.2 Treatment‐emergent adverse events (TEAEs)

A total of 24 patients (96%) were reported with TEAEs (208 cases), for 23 patients (92%) TEAEs were considered related to the study drug (123 cases). Serious TEAEs (TESAE) were documented in 9 patients (36%, 18 cases) which were drug related in 5 patients (20%, 13 cases). Fourteen patients (56%) presented with TEAEs of CTCAE toxicity grade 3/4 (32 cases) and 52% of patients had CTCAE toxicity grade 3/4 TEAEs related to study drug (24 cases).

The most common TEAEs (any grade; Table 4) were fatigue (36%, n = 9), nausea (28%, n = 7), alanine aminotransferase increased (24%, n = 6), aspartate aminotransferase increased (24%, n = 6), depression (24%, n = 6), and dizziness (24%, n = 6). The most common TEAEs of grade 3/4 were alanine aminotransferase increased (20%, n = 5), aspartate aminotransferase increased (16%, n = 4), pruritus (8%, n = 2), and blood pressure increased (8%, n = 2) as further detailed in Table 4.

**TABLE 4:** Treatment‐emergent adverse events (≥10% of patients, any grade and ≥1 patient, grade 3/4; SAF)

MedDRA Preferred Term, n (%)	All grades^a^	Grade 3/4
Total	24 (96.0)	14 (56.0)
Fatigue	9 (36.0)	0 (0)
Nausea	7 (28.0)	1 (4.0)
Alanine aminotransferase increased	6 (24.0)	5 (20.0)
Aspartate aminotransferase increased	6 (24.0)	4 (16.0)
Dizziness	6 (24.0)	1 (4.0)
Depression	6 (24.0)	1 (4.0)
Vomiting	5 (20.0)	0 (0)
Anxiety	5 (20.0)	0 (0)
Diarrhea	4 (16.0)	0 (0)
Viral upper respiratory tract infection	4 (16.0)	0 (0)
Decreased appetite	4 (16.0)	0 (0)
Pruritus	4 (16.0)	2 (8.0)
Blood pressure increased	3 (12.0)	2 (8.0)
Hyperglycemia	3 (12.0)	1 (4.0)
Muscle spasms	3 (12.0)	0 (0)
Dysgeusia	3 (12.0)	0 (0)
Headache	3 (12.0)	0 (0)
Paraesthesia	3 (12.0)	0 (0)
Mood altered	3 (12.0)	1 (4.0)
Palmar‐plantar erythrodysaesthesia syndrome	3 (12.0)	1 (4.0)
Photosensitivity reaction	3 (12.0)	1 (4.0)
Confusional state	2 (8.0)	1 (4.0)
Cataract	1 (4.0)	1 (4.0)
Hepatotoxicity	1 (4.0)	1 (4.0)
Infection	1 (4.0)	1 (4.0)
Blood pressure abnormal	1 (4.0)	1 (4.0)
Vitamin D deficiency	1 (4.0)	1 (4.0)
Cognitive disorder	1 (4.0)	1 (4.0)
Syncope	1 (4.0)	1 (4.0)
Mania	1 (4.0)	1 (4.0)
Breast inflammation	1 (4.0)	1 (4.0)
Erythema	1 (4.0)	1 (4.0)
Hypertension	1 (4.0)	1 (4.0)

Note

SAF population (n = 25). AEs with a relative frequency of ≥10% of total population (any grades) and for ≥1 patient (grade 3/4) are displayed. Patients are sorted according to their absolute frequency (AEs of all grades). Displayed are treatment‐emergent AEs. AEs were classified as treatment emergent if they occurred or worsened during the on‐treatment phase (defined as time period from day of first dose of buparlisib to 30 d after last dose of buparlisib). For AEs (MedDRA v20.0 preferred terms) occurring more than once per patient, the AE with the highest severity grade was used in the analysis.

Abbreviations: MedDRA, Medical Dictionary for Regulatory Activities.

^a^ Multiple entries per patient possible.

#### 4.4.3 Specific safety event categories

The SECs, used as categorization of the reported TEAEs, included gastrointestinal events (56%), psychiatric/ mood disorders (48%), skin rash/ hypersensitivity (44%), cardiovascular events (40%), liver toxicity (32%), and hyperglycemia (16%).

#### 4.4.4 Fatal events

Overall, 15 patients (60%) died during the study (PD, n = 13; acute liver failure, n = 1; unknown, n = 1). Death was reported as fatal TEAE in one patient (PD) during treatment phase. None of the deaths were attributable to study treatment.

#### 4.4.5 Depression and anxiety—PHQ‐9 and GAD‐7 Questionnaires

At baseline (N = 25), the median total score on the PHQ‐9 questionnaire was 2.0 (min‐max: 0‐9) and the median total score on the GAD‐7 questionnaire was 1.0 (min‐max: 0‐6). The median total scores of PHQ‐9 and GAD‐7 did not change considerably during the treatment phase (minimal observed median: 0.0, maximal observed median: 3.0). Unchanged severity grades were reported for most patients (PHQ‐9: n = 16; GAD‐7: n = 16). Regarding PHQ‐9 and GAD‐7, 9 patients experienced worsening of their health status (at least one category) from baseline to worst on treatment (PHQ‐9: “none” to “mild”: n = 3, “none” to “moderate”: n = 3, “none” to “severe”: n = 1, “mild” to “moderate”: n = 2; GAD‐7: “none” to “mild”: n = 6, and “none” to “severe”: n = 3).

## 5 DISCUSSION

Preclinical studies suggested synergistic effects of PI3K inhibition and ET in HR^+^ BC patients, particularly in tumors with biological indicators of pathway activation such as *PIK3CA* mutations.^25^, ^26^, ^44^ Against this background we designed the study PIKTAM to prospectively evaluate efficacy and safety of a pan‐PI3K inhibitor, buparlisib, in combination with tamoxifen in HR^+^ mBC patients stratified by biomarkers of pathway activation, determined both in tumor tissue and cfDNA. The study mainly involved community oncology practices and centers and thus prospectively explored the feasibility of precision oncology strategies in mBC in a real‐world setting.

Unfortunately, study enrolment had to be prematurely terminated by the sponsor after re‐evaluation of the safety and risk/benefit profile of buparlisib based on emerging results of the BELLE‐2 study.^19^ At that time, only 25 of 99 planned patients had been recruited. Accordingly, PIKTAM could not be conducted following the original study plan, and all analyses are to be viewed exploratory.

The median PFS obtained in the PIKTAM trial (6.1 months) was comparable to the median PFS in the BELLE‐2 trial^19^ (6.9 months, buparlisib + fulvestrant arm), and was numerically longer than in the BELLE‐3 trial^20^ (3.9 months, buparlisib + fulvestrant arm). This may be ascribed to differences in baseline characteristics, such as pretreatment of patients with a mTOR inhibitor (in the metastatic setting) in the BELLE‐3 trial. Still, the 6‐month PFS rate of 33.33% observed in the PIKTAM study is comparable to the BELLE‐3 trial (31% in buparlisib + fulvestrant arm).^20^ Hence, our study lends further support to the therapeutic strategy combining inhibitors of the PI3K pathway with antihormonal agents in patients with HR^+^ HER2^−^ mBC.

A particularly interesting signal was derived from comparative analyses of the predefined molecular subgroups of PIKTAM, which support the concept of precision oncology in HR^+^ mBC: In patients with tumors harboring oncogenic *PIK3CA* mutations, a clear trend of improved outcomes with buparlisib/tamoxifen treatment was observed. This was true for the primary study endpoint, 6‐month PFS rate (62.50%), as well as for median PFS (8.7 months), ORR (40%), and DCR (80%), while OS data were still immature. These results are in line with preclinical and retrospective clinical data indication increased sensitivity of PIK3CA‐mutated breast cancer to ET^45^, ^46^ and combined estrogen deprivation and PI3K inhibitors.^25^ Together with buparlisib's increased activity in *PIK3CA* mutant cell lines in vitro,^23^ there is a clear rationale for prospective testing of the clinical utility of *PIK3CA* mutations as predictive biomarker.

Currently, there is much interest in exploring alternative formats of genomic biomarkers, particularly testing of circulating free DNA, which also contains tumor cell‐derived DNA (ctDNA). In BELLE‐2,^19^ detection of *PIK3CA* mutations in ctDNA clearly outperformed analysis of *PIK3CA* mutations or loss of PTEN expression in archival tumor specimens. This observation was primary explained by the fact that ctDNA analyzed immediately prior to treatment initiation is more suited to reflect the current status of a systemic malignancy than historical tissue samples.^19^ However, the BELLE‐3 trial^20^ showed improved PFS with study medication in patients with *PIK3CA* mutations, as obtained from both cfDNA and tumor tissue analysis. In our study, *PIK3CA* mutation analysis of tumor‐derived DNA provided a strong signal as a positive predictive biomarker for benefit from buparlisib/tamoxifen combination. Loss of PTEN expression alone had no such effect, but interpretation clearly is hampered by the low patient number. Interestingly, combining both markers in an exploratory “PI3K activation” group maintained and possibly even expanded the predictive signal.

With the caveat of low patient numbers, in PIKTAM *PIK3CA* mutation detection in ctDNA did not seem to add predictive power as compared to tumor tissue‐based biomarker detection.

Recently, isoform‐specific PI3K inhibitors have emerged as new treatment option in HR + mBC with a more favorable toxicity profile than pan‐class I PI3K inhibitors. In the SOLAR‐1 study, the α‐specific PI3K inhibitor alpelisib plus fulvestrant was compared to fulvestrant alone. The combination was clearly superior in patients with *PIK3CA*‐mutated HR + mBC as detected in tumor DNA (median PFS: 11 months vs 5.7 months; ORR: 36% vs 16%) or in cfDNA (median PFS: 10.9 months vs 3.7 months; ORR: not reported).^47^, ^48^ These recent findings further support the precision oncology concept prospectively tested in our PIKTAM study.

In summary, the PIKTAM study confirmed the feasibility of the implementation of a precision oncology concept for mBC patients in a network involving community oncology practices and a major academic comprehensive cancer center. Due to premature study termination for altered risk‐benefit assessment of buparlisib, we were unable to complete the original study plan. However, our analyses of prespecified, biomarker‐defined patient subgroups provide important evidence in support of the clinical utility of *PIK3CA* mutations as predictive biomarkers for PI3K‐targeting therapies in HR + mBC.

## SPONSOR

Universitätsklinikum Essen, Deutschland.

## Supporting information

Supplementary Material

## Data Availability

Clinical data were documented in electronic Case Report Forms (eCRFs; *iostudy office edc*, iOMEDICO) and are the property of iOMEDICO. The data are not publicly available due to them containing information that could compromise patient and/or research participant privacy/ consent.
