# Supplementary material for: Buparlisib in combination with tamoxifen in pretreated patients with hormone receptor‐positive, HER2‐negative advanced breast cancer molecularly stratified for PIK3CA mutations and loss of PTEN expression
Source: Cancer Med. 2020 Apr 30;9(13):4527–39. doi: 10.1002/cam4.3092 (PMC7333856; doi:10.1002/cam4.3092)
Supplement: Supplementary file 1 — Supplementary Material [file CAM4-9-4527-s001.docx]

Supplement 1

Mutation status of *PIK3CA* exon 20 in tumor sample compared to cfDNA mutation status (at baseline)

| *PIK3CA* exon 20 mutation status | cfDNA mutation (N=4) | cfDNA wild-type (N=14) |
| --- | --- | --- |
| tumor sample wild-type, n (%) | 1 (25.0) | 12 (85.7) |
| tumor sample mutation, n (%) | 3 (75.0) | 2 (14.3) |

Supplement 2

*PIK3CA* Mutation analysis (per patient): tumor vs cfDNA mutation status

| Biomarker stratification group | Included in cfDNA subgroup analysis | Treatment Flag | *PIK3CA* cfDNA mutation status | *PIK3CA* tumor sample mutation status | |
| --- | --- | --- | --- | --- | --- |
|  |  |  | Exon 20 | Exon 9 | Exon 20 |
| *PIK3CA* wt / PTEN preserved | Y | Y | no mutation | no mutation | no mutation |
| *PIK3CA* wt / PTEN preserved | Y | Y | no mutation | no mutation | no mutation |
| *PIK3CA* mut / PTEN preserved | N | N | no mutation | mutation | no mutation |
| *PIK3CA* mut / PTEN preserved | N | Y | no mutation | mutation | no mutation |
| *PIK3CA* mut or wt / PTEN loss | Y | Y | no mutation | no mutation | no mutation |
| *PIK3CA* wt / PTEN preserved | Y | Y | no mutation | no mutation | no mutation |
| *PIK3CA* wt / PTEN preserved | N | N | no mutation | no mutation | no mutation |
| *PIK3CA* wt / PTEN preserved | Y | Y | no mutation | no mutation | no mutation |
| *PIK3CA* mut / PTEN preserved | Y | Y | no mutation | no mutation | mutation |
| *PIK3CA* wt / PTEN preserved | Y | Y | no mutation | no mutation | no mutation |
| *PIK3CA* wt / PTEN preserved | Y | Y | no mutation | no mutation | no mutation |
| *PIK3CA* mut / PTEN preserved | Y | Y | mutation | no mutation | mutation |
| *PIK3CA* wt / PTEN preserved | Y | Y | no mutation | no mutation | no mutation |
| *PIK3CA* wt / PTEN preserved | Y | Y | no mutation | no mutation | no mutation |
| *PIK3CA* mut / PTEN preserved | Y | Y | no mutation | no mutation | mutation |
| *PIK3CA* mut / PTEN preserved | Y | Y | mutation | no mutation | mutation |
| *PIK3CA* mut / PTEN preserved | Y | Y | mutation | no mutation | mutation |
| *PIK3CA* mut or wt / PTEN loss | N | N | mutation | no mutation | mutation |
| *PIK3CA* wt / PTEN preserved | Y | Y | mutation | no mutation | no mutation |
| *PIK3CA* mut / PTEN preserved | N | Y | no mutation | mutation | no mutation |
| *PIK3CA* mut / PTEN preserved | N | N | mutation | no mutation | mutation |
| *PIK3CA* wt / PTEN preserved | Y | Y | no mutation | no mutation | no mutation |
| *PIK3CA* wt / PTEN preserved | Y | Y | no mutation | no mutation | no mutation |
| *PIK3CA* wt / PTEN preserved | Y | Y | no mutation | no mutation | no mutation |
| *PIK3CA* mut / PTEN preserved | N | Y | no mutation | mutation | no mutation |

# Supplement 3: 6-month PFS rate by biomarker group (mITT)


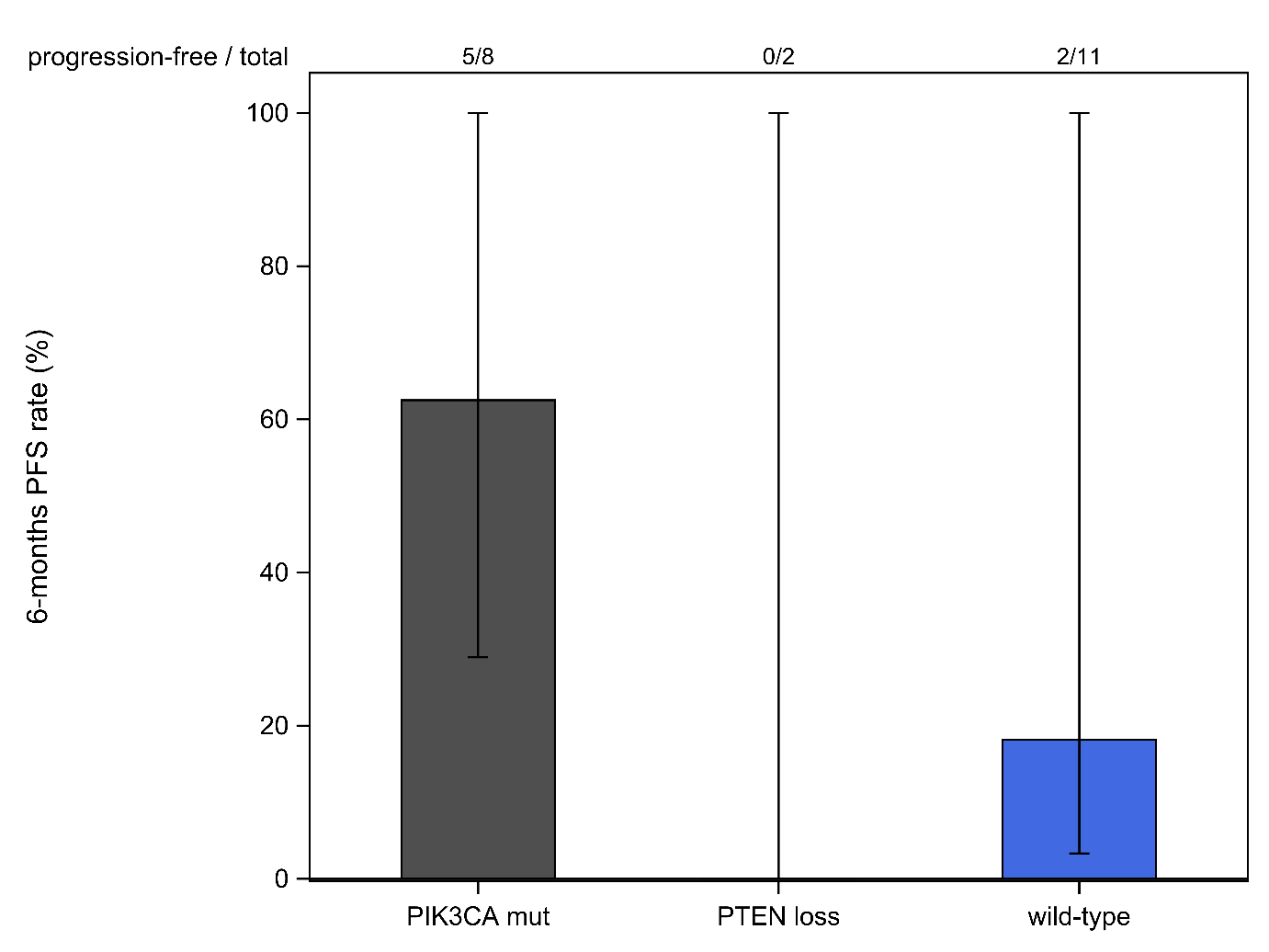


Error bars represent one-sided 95% confidence intervals

# Supplement 4: Overall survival

### Overall survival in total patient population (SAF)


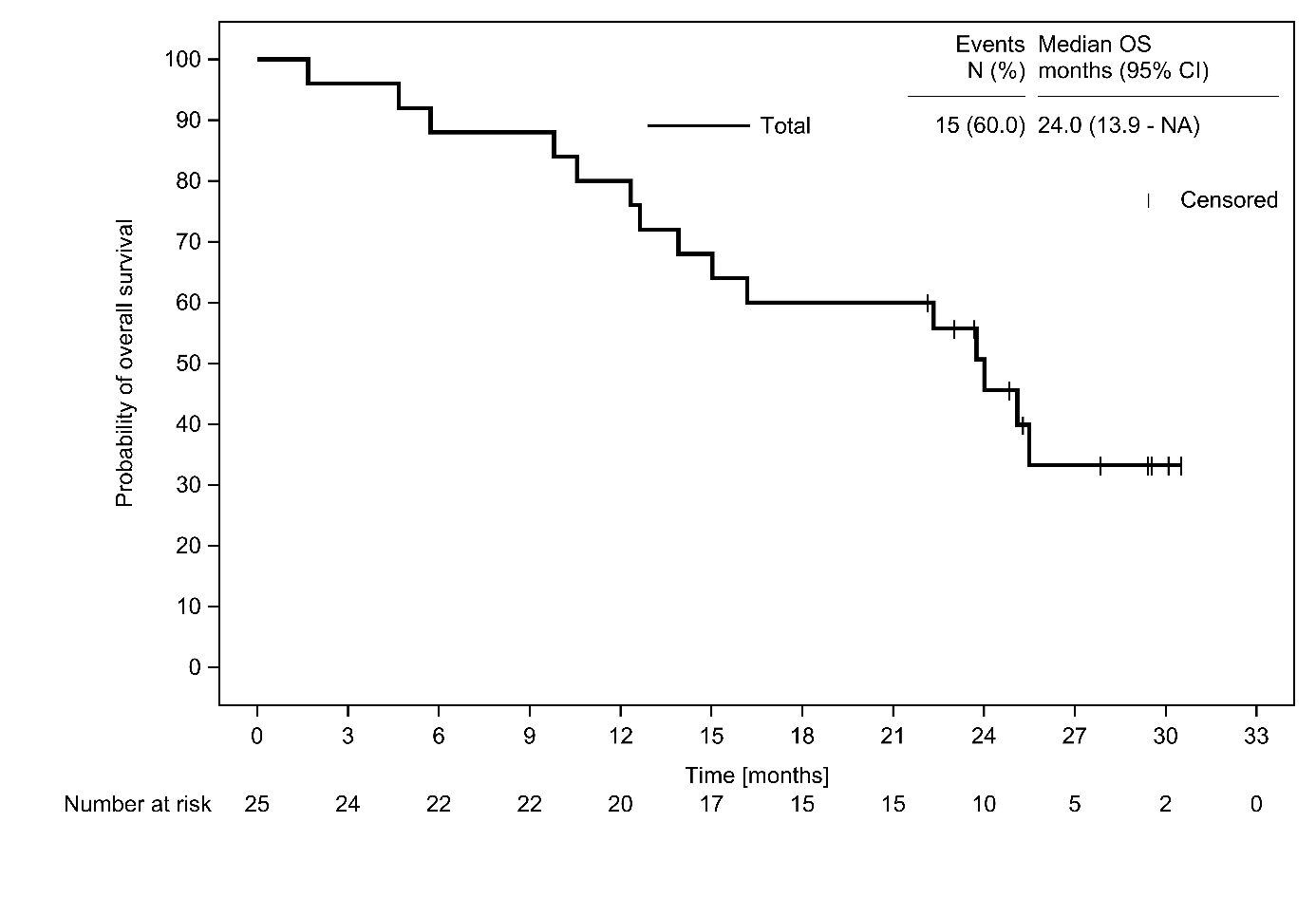


### Overall survival by biomarker stratification group (SAF)


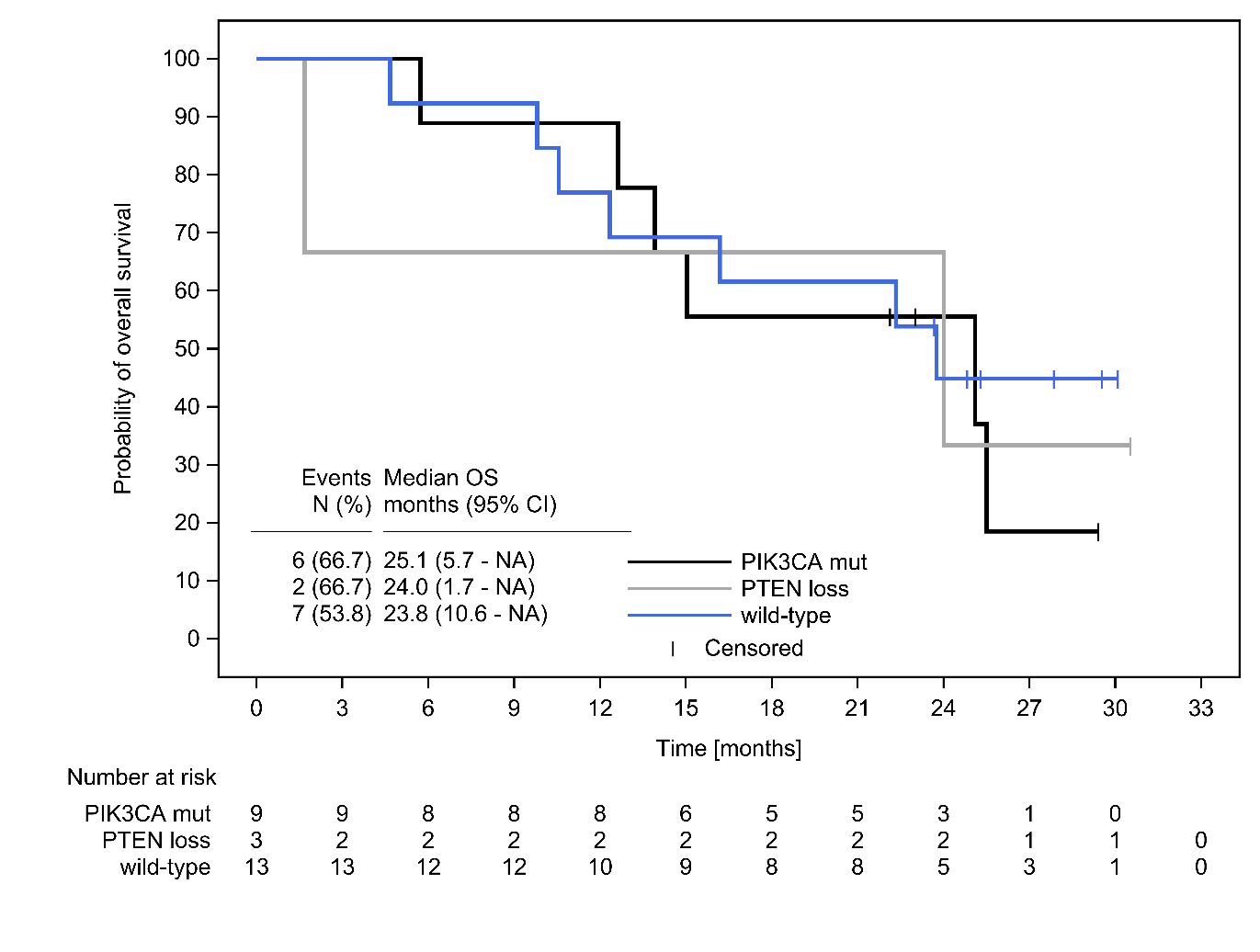


*PIK3CA* mut = *PIK3CA* mut / PTEN preserved; PTEN loss = *PIK3CA* mut or wt / PTEN loss; wild-type = *PIK3CA* wt / PTEN preserved

### Overall survival in PI3K activated and PI3K non-activated subgroup

The median OS was 23.8 months in the “PI3K non-activated” (N=13) subgroup and 24.0 months in the “PI3K activated” subgroup (N=12). The 1- year and 2-year OSRs in the subgroup “PI3K non-activated” were 76.9% (95%CI 44.2 – 91.9) and 44.9% (95%CI 17.7 – 69.0). For the subgroup “PI3K activated”, the corresponding values were 83.3% (95%CI 48.2 – 95.6) and 58.3% (27.0 – 80.1), respectively.

### Overall survival by cfDNA mutation status

According to *PIK3CA* cfDNA mutation status, median OS was 25.5 months (95% CI 12.3 – NA) both in the total population (N=18) and the “*PIK3CA* cfDNA wild-type” subgroup (N=14). In the “*PIK3CA* cfDNA mutation” subgroup (N=4), the median OS had not yet been reached (95% CI 5.7 – NA). The 1-year and 2-year OSRs in the total population were 77.8% (95% CI 51.1 – 91.0) and 60.0% (95% CI 33.7 – 78.7). In the “*PIK3CA* cfDNA mutation” subgroup, the 1-year and 2-year OSRs were each 50.0% (95% CI 5.8 – 84.5) and for the “*PIK3CA* cfDNA wild-type subgroup”, these were 85.7% (95% CI 53.9 – 96.2) and 63.5% (95% CI 33.1 – 83.0), respectively.

# Supplement 5: Response rates

In the PI3K non-activated subgroup (N=9), ORR was 0% (n=0, 95% CI 0.0 – 33.6) and DCR was 33.3% (n=3, 95% CI 7.5 – 70.1). In the PI3K activated subgroup (N=7), ORR was 28.6% (n=2, 95% CI 3.7 – 71.0) and DCR was 57.1% (n=4, 95% CI 18.4 – 90.1). According to *PIK3CA* cfDNA mutation status, ORR and DCR were 18.2% (n=2, 95% CI 2.3 – 51.8) and 54.5% (n=6, 95% CI 23.4 – 83.3) in the total population (N=11), 50.0% (n=1, 95% CI 1.3 – 98.7) and 50% (n=1, 95% CI 1.3 – 98.7) in the subgroup “*PIK3CA* cfDNA mutation” (N=2) and 11.1% (n=1, 95% CI 0.3 – 48.2) and 55.6% (n=5, 95% CI 21.2 – 86.3) in the “*PIK3CA* cfDNA wild-type” subgroup (N=9).
